# Integrated Transcriptomic Analysis Suggests a C1Q-Related Macrophage–Fibroblast Signaling Axis in Keloids

**DOI:** 10.3390/ijms27115140

**Published:** 2026-06-05

**Authors:** Yutong Yuan, Yuanbo Liu, Ningbei Yin, Shan Zhu, Nuo Si

**Affiliations:** Plastic Surgery Hospital, Chinese Academy of Medical Sciences, Peking Union Medical College, No. 33 Ba-Da-Chu Road, Beijing 100144, China; y504071842@163.com (Y.Y.); yb5886@163.com (Y.L.); yinningbei@psh.pumc.edu.cn (N.Y.)

**Keywords:** keloid, bioinformatics analysis, cell–cell communication, single-cell transcriptomics, macrophage, C1Q, LRP1

## Abstract

Keloids are fibroproliferative scars characterized by immune dysregulation, persistent fibroblast activation, and excessive extracellular matrix deposition. The mechanisms by which macrophage functional states regulate pathological fibroblast activation remain incompletely understood. Bulk RNA-seq and single-cell RNA-seq datasets were integrated to characterize the immune–fibrotic microenvironment of keloids and infer candidate macrophage–fibroblast communication axes. Candidate findings were further assessed by tissue immunofluorescence and preliminary in vitro phenotypic evaluation using C1Q stimulation and RAP intervention in keloid fibroblasts. Bulk transcriptomic analysis revealed coordinated activation of immune-related and fibrotic remodeling programs in keloids. Single-cell analysis identified activated profibrotic fibroblast states and a macrophage population with M2-like features. Ligand–target inference nominated *C1QB* as a candidate macrophage-associated ligand transcript potentially involved in fibroblast activation, while low-density lipoprotein receptor-related protein 1 (LRP1), a biologically plausible receptor candidate, was enriched in activated fibroblast states. Tissue immunofluorescence showed the coexistence of C1QB^+^ macrophages and LRP1^+^ fibroblasts in keloid lesions. In vitro phenotypic assessment using the intact C1Q complex showed that C1Q increased *ACTA2* expression in primary keloid fibroblasts, whereas RAP attenuated this effect; *COL1A1* and *POSTN* showed no consistent induction. Together, these findings support a candidate C1Q-related macrophage–fibroblast communication model and provide a preliminary framework for understanding immune-driven fibroblast activation during keloid progression, with implications for future exploration of potential intervention strategies.

## 1. Introduction

Keloids are pathological scars that arise from dysregulated wound repair and are characterized by progressive extension beyond the original wound boundaries into adjacent normal skin. Their histopathological features include persistent fibroblast activation and excessive extracellular matrix (ECM) deposition with aberrant matrix remodeling; however, the molecular mechanisms that drive their initiation and sustained progression remain incompletely defined. Emerging evidence indicates that keloid pathogenesis is not attributable solely to intrinsic fibroblast abnormalities. Compared with normal skin, keloid tissues exhibit marked alterations in the abundance and states of immune cells, including macrophages, T cells, and mast cells, together with substantial changes in cytokine expression profiles. Together, these findings suggest that immune–stromal microenvironmental remodeling is central to keloid formation and persistence [1,2].

Recent studies have mapped the cellular landscape of keloids, revealing marked heterogeneity of both fibroblasts and immune cell subsets within keloid lesions [3]. Among immune cells, macrophages have emerged as key regulators of the fibrotic microenvironment in keloids. Keloid-associated macrophages predominantly exhibit M2-like polarization a state closely associated with tissue repair, matrix deposition, and fibrotic progression [4], suggesting that these cells may act not merely as inflammatory bystanders but as upstream regulators of pathological fibroblast activation. Notably, macrophage subsets characterized by high expression of C1Q and its component subunits have been identified in keloid lesions, implicating C1Q-related signaling in aberrant scar formation [5,6]. But the paracrine function of this C1Q-high macrophage subset, as well as its specific ligand–receptor axis regulating fibroblast activation in keloids, remains completely uncharacterized.

C1Q is the core recognition molecule of the classical complement pathway, and recent studies across multiple fibrotic diseases (including pulmonary fibrosis, muscle regenerative fibrosis, and liver fibrosis) have demonstrated that macrophage-derived C1Q is not only a phenotypic marker of macrophage subsets, but also a biologically active paracrine signal regulating stromal cell activation and tissue remodeling [7,8,9]. The intact functional C1Q complex is a multimeric protein composed of six heterotrimers, each containing C1QA, C1QB, and C1QC chains [10]. Therefore, C1QB expression in single-cell transcriptomic data should be interpreted primarily as a marker of a C1Q-high macrophage state, whereas the functional paracrine effect is expected to be mediated by the assembled C1Q protein complex. In parallel, low-density lipoprotein receptor-related protein 1 (LRP1, also known as CD91) is a multifunctional endocytic and signaling receptor that regulates diverse signaling pathways related to platelet-derived growth factor receptor (PDGFR), transforming growth factor beta (TGF-β), and integrins, and has been implicated in myofibroblast-like transition and disturbed ECM homeostasis [11,12,13]. Importantly, C1Q has been shown to directly bind LRP1, and this interaction can be competitively inhibited by receptor-associated protein (RAP), a broad-spectrum inhibitor of the low-density lipoprotein (LDL) receptor family [14,15]. Together, these findings suggest that macrophage-derived C1Q may regulate fibroblast activation through LRP1 in fibrotic diseases, but this axis has not been explored in keloid pathology.

Although previous studies have suggested the coexistence of C1Q-high macrophage subsets and profibrotic stromal cell states in keloids, there is no direct evidence linking C1Q-related signaling to pathological fibroblast activation in keloids, and the specific ligand–receptor axis and functional effects remain unclear [16,17]. Here, we integrated bulk RNA-seq and single-cell RNA-seq analyses to systematically characterize the immune–stromal microenvironment of keloids, with a focus on cellular states and communication axes associated with aberrant fibroblast activation. We further provide preliminary evidence supporting a candidate communication model in which C1QB-high profibrotic macrophage states may be linked to LRP1-positive activated fibroblast states through C1Q-related signaling. These findings provide a macrophage–fibroblast crosstalk-centered framework for understanding immune-driven fibroblast activation during keloid progression and may inform future studies exploring potential intervention strategies.

## 2. Results

### 2.1. Bulk Transcriptomic Profiling Reveals Coordinated Immune Activation and ECM-Associated Fibrotic Remodeling in Keloids

**Figure 1:**
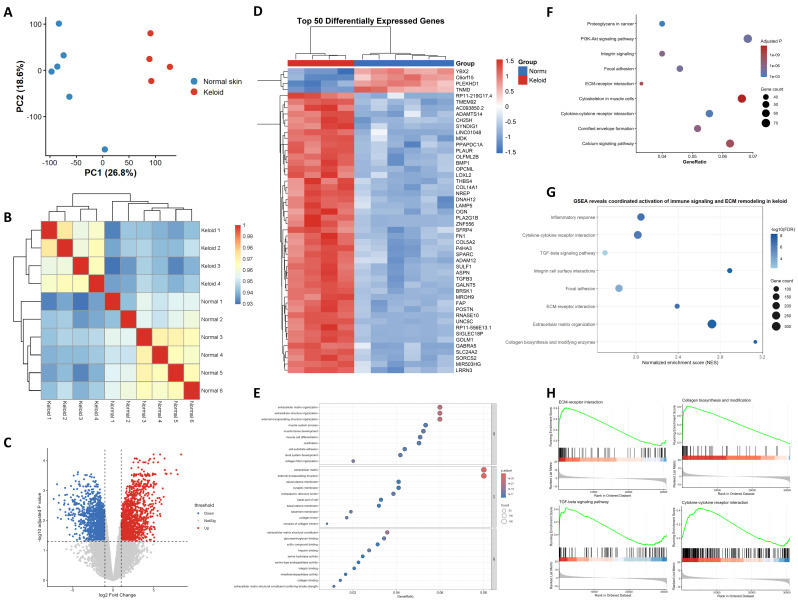
Bulk RNA-seq analysis reveals enhanced immune activation and ECM remodeling-related transcriptional programs in keloid tissue. (**A**) Principal component analysis of bulk RNA-seq samples from normal skin and keloid tissue. Each dot represents one sample; (**B**) transcriptome-wide sample correlation heatmap of normal skin and keloid tissue. Colors indicate Pearson correlation coefficients; (**C**) volcano plot showing differentially expressed genes between keloid tissue and normal skin. Red indicates significantly upregulated genes, blue indicates significantly downregulated genes, and gray indicates non-significant genes; (**D**) heatmap of the top 50 differentially expressed genes. Expression values were scaled by row; (**E**) GO enrichment analysis of differentially expressed genes. Dot size indicates the number of enriched genes, and color indicates the adjusted *p* value; (**F**) KEGG pathway enrichment analysis of differentially expressed genes. Dot size indicates the number of enriched genes, and color indicates the adjusted *p* value; (**G**) GSEA summary plot showing representative pathways enriched in keloid tissue. The x-axis indicates the NES, dot size indicates gene set size, and color indicates enrichment significance; (**H**) GSEA enrichment plots of representative pathways, including ECM–receptor interaction, cytokine–cytokine receptor interaction, TGF-beta signaling pathway, and collagen biosynthesis and modification. Differentially expressed genes were defined using an adjusted *p* value < 0.05 and |log_2_FC| > 1. Abbreviations: ECM, extracellular matrix; GO, Gene Ontology; GSEA, gene set enrichment analysis; KEGG, Kyoto Encyclopedia of Genes and Genomes; NES, normalized enrichment score.

Bulk RNA-seq analysis revealed a distinct transcriptomic profile in keloid tissues compared with normal skin. Principal component analysis showed clear separation between the two groups, and sample correlation analysis confirmed group-specific clustering with high within-group consistency (Figure 1A,B). A total of 2941 differentially expressed genes (DEGs) were identified in keloids, including 1437 upregulated and 1504 downregulated genes (Figure 1C; Supplementary Table S2). The top DEGs further distinguished keloids from normal skin, with marked upregulation of ECM deposition- and tissue remodeling-related genes, including *FN1*, *SPARC*, *POSTN*, *FAP*, *TGFB3*, and *LOXL2* (Figure 1D).

Functional enrichment analysis showed that keloid-associated DEGs were significantly enriched in ECM remodeling-related biological processes, including extracellular matrix organization. Kyoto Encyclopedia of Genes and Genomes (KEGG) analysis highlighted enrichment of ECM–receptor interaction and cytokine–cytokine receptor interaction pathways, indicating activation of both matrix remodeling and cytokine-mediated signaling programs (Figure 1E,F and Figure S1A,B; Supplementary Tables S3 and S4). Consistently, gene set enrichment analysis (GSEA) demonstrated enhanced enrichment of ECM–receptor interaction, collagen biosynthesis and modification, TGF-β signaling, and cytokine–cytokine receptor interaction pathways in keloid tissues (Figure 1G,H).

**Figure 2:**
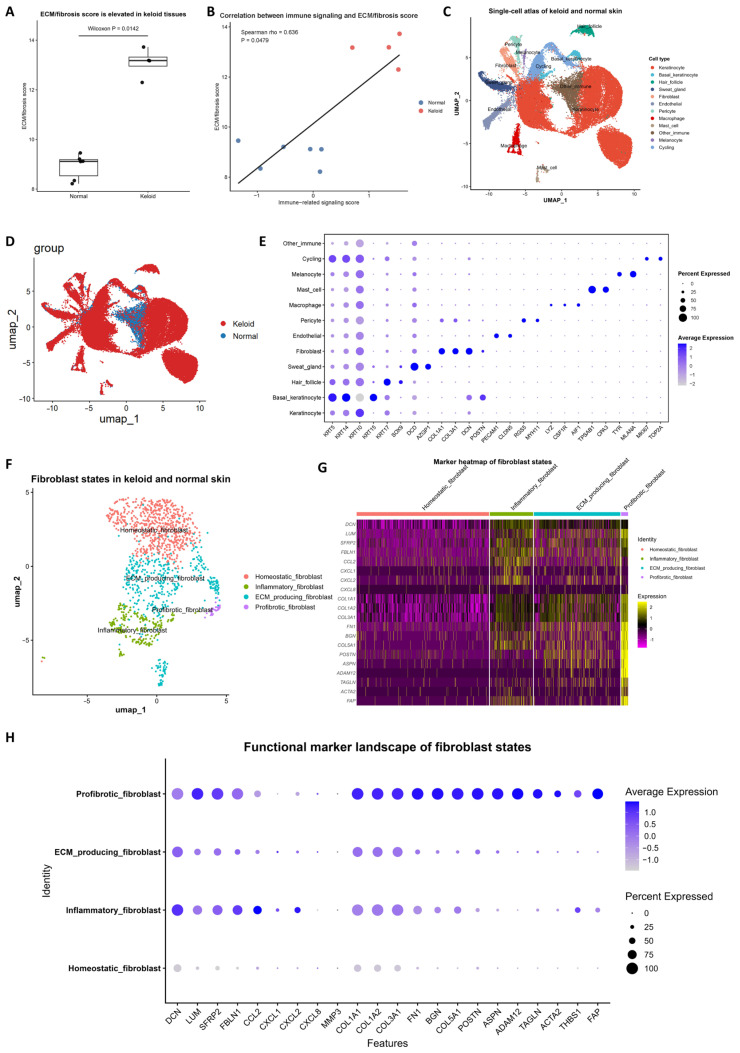
Bulk transcriptomic scoring and single-cell atlas define the immune–fibrotic microenvironment and fibroblast heterogeneity in keloid. (**A**) Comparison of ECM/fibrosis scores between normal skin and keloid tissue; (**B**) correlation analysis between immune-related signaling score and ECM/fibrosis score; (**C**) UMAP visualization of the integrated single-cell transcriptomic atlas annotated by major cell types; (**D**) UMAP distribution of cells derived from normal skin and keloid tissue in the integrated single-cell atlas; (**E**) expression patterns of representative marker genes across major cell types. Dot size indicates the proportion of positive cells, and color indicates the average expression level; (**F**) UMAP visualization and state annotation of reclustered fibroblast populations; (**G**) heatmap showing representative marker genes across fibroblast states; (**H**) expression patterns of functional marker genes across fibroblast states. Dot size indicates the proportion of positive cells, and color indicates the average expression level. Group differences in (**A**) were assessed using the Wilcoxon rank-sum test. Correlation in (**B**) was assessed using Spearman correlation analysis. Abbreviations: ECM, extracellular matrix; UMAP, uniform manifold approximation and projection.

At the sample level, the ECM/fibrosis score was significantly higher in keloids than in normal skin (Figure 2A). Moreover, the immune-related signaling score positively correlated with the ECM/fibrosis score (rho = 0.636, *p* = 0.0479), suggesting that immune activation is closely associated with enhanced ECM remodeling and fibrotic transcriptional programs (Figure 2B). Together, these findings indicate that keloids exhibit coordinated immune activation and ECM-associated fibrotic remodeling at the bulk transcriptomic level.

### 2.2. Single-Cell Transcriptomic Profiling Defines the Cellular Landscape of the Keloid Immune–Fibrotic Microenvironment and Reveals Fibroblast Heterogeneity

To define the cellular basis of the keloid immune–fibrotic microenvironment, we analyzed single-cell RNA-seq data from keloid and normal skin tissues. After quality control, 47,451 high-quality cells were retained from 71,050 captured cells (Figure S1C). Unsupervised clustering and Uniform Manifold Approximation and Projection (UMAP) visualization identified major skin cell populations, including keratinocytes, basal keratinocytes, hair follicle cells, sweat gland cells, fibroblasts, endothelial cells, pericytes, macrophages, mast cells, melanocytes, cycling cells, and other immune cells (Figure 2C and Figure S1D–G; Supplementary Table S5).

Group-based visualization showed both shared and condition-biased cellular distributions between normal skin and keloid tissues, suggesting disease-associated remodeling of cellular composition and transcriptional states (Figure 2D). Cell-type annotations were supported by canonical lineage markers, including *COL1A1*, *COL3A1*, *DCN*, and *POSTN* for fibroblasts; *LYZ*, *CSF1R*, and *AIF1* for macrophages; *KRT5*, *KRT14*, *KRT10*, and *KRT15* for keratinocyte lineages; *KRT17* and *SOX9* for hair follicle stem/progenitor cells; *DCD* and *AZGP1* for sweat gland cells; *PECAM1* and *CLDN5* for endothelial cells; *RGS5* and *MYH11* for pericytes; *TPSAB1* and *CPA3* for mast cells; *TYR* and *MLANA* for melanocytes; and *MKI67* and *TOP2A* for cycling cells (Figure 2E).

Given the prominent ECM remodeling and fibrotic programs observed in bulk transcriptomic analysis, we next focused on fibroblasts. After subclustering and removal of non-fibroblast contaminating clusters, fibroblasts were resolved into four states: homeostatic, inflammatory, ECM-producing, and profibrotic fibroblasts (Figure 2F). Homeostatic fibroblasts preferentially expressed *DCN*, *LUM*, *SFRP2*, and *FBLN1*; inflammatory fibroblasts were enriched for *CCL2*, *CXCL1*, *CXCL2*, and *CXCL8*; ECM-producing fibroblasts showed high expression of *COL1A1*, *COL1A2*, *COL3A1*, *FN1*, and *BGN*; and profibrotic fibroblasts were characterized by fibrosis and myofibroblast activation-associated genes, including *POSTN*, *ASPN*, *ADAM12*, *TAGLN*, *ACTA2,* and *FAP* (Figure 2G,H).

### 2.3. Functional Profiling Identifies ECM-Producing and Profibrotic Fibroblasts as Major Fibrosis-Associated Populations in Keloids

**Figure 3:**
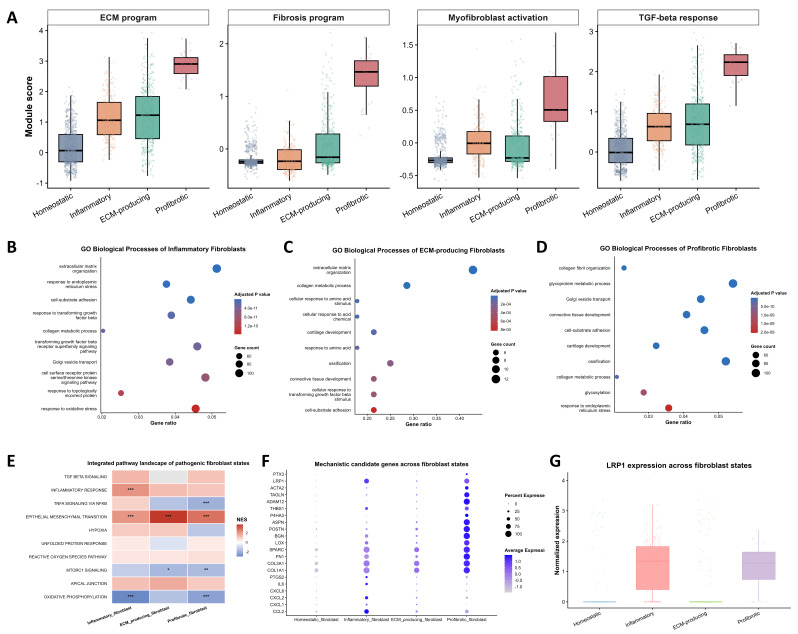
Functional profiling identifies ECM-producing and profibrotic fibroblasts as major fibrosis-associated fibroblast states in keloid. (**A**) Module scores for ECM program, fibrosis program, myofibroblast activation, and TGF-beta response across fibroblast states; (**B**) GO biological process enrichment analysis of Inflammatory_fibroblast. Dot size indicates the number of enriched genes, and color indicates the adjusted *p* value; (**C**) GO biological process enrichment analysis of ECM_producing_fibroblast. Dot size indicates the number of enriched genes, and color indicates the adjusted *p* value; (**D**) GO biological process enrichment analysis of Profibrotic_fibroblast. Dot size indicates the number of enriched genes, and color indicates the adjusted *p* value; (**E**) Hallmark pathway enrichment heatmap across fibroblast states. Colors indicate normalized enrichment scores; (**F**) expression patterns of key functional genes across fibroblast states. Dot size indicates the proportion of positive cells, and color indicates the average expression level; (**G**) comparison of *LRP1* expression across fibroblast states. Significance levels are indicated as follows: * *p* < 0.05; ** *p* < 0.01; *** *p* < 0.001. Abbreviations: ECM, extracellular matrix; GO, Gene Ontology.

Functional profiling revealed distinct state-specific programs across fibroblast populations. Profibrotic fibroblasts exhibited the highest ECM, fibrosis, myofibroblast activation, and TGF-β response scores, consistent with a highly activated profibrotic phenotype. ECM-producing fibroblasts were primarily characterized by enhanced ECM-related programs but showed weaker myofibroblast activation, suggesting a predominant role in matrix synthesis and deposition. Inflammatory fibroblasts also displayed moderate ECM- and TGF-β-related activity, but had lower fibrosis and myofibroblast activation scores than profibrotic fibroblasts (Figure 3A).

Functional enrichment analysis further supported this fibroblast state specialization. Inflammatory fibroblasts were enriched for inflammatory and stress-related processes, ECM-producing fibroblasts for extracellular matrix organization, and profibrotic fibroblasts for cell–substrate adhesion, connective tissue development, and endoplasmic reticulum stress response (Figure 3B–D). Hallmark pathway analysis confirmed this functional divergence, with inflammatory fibroblasts showing inflammatory response enrichment, whereas ECM-producing and profibrotic fibroblasts showed stronger epithelial–mesenchymal transition and stromal remodeling signatures (Figure 3E and Figure S1H–J). Consistent with these programs, inflammatory fibroblasts highly expressed *CCL2*, *CXCL1*, *CXCL2*, *CXCL8*, *IL6*, and *PTGS2*; ECM-producing fibroblasts highly expressed *COL1A1*, *COL3A1*, *FN1*, *SPARC*, *LOX*, and *BGN*; and profibrotic fibroblasts were marked by *POSTN*, *ASPN*, *ADAM12*, *TAGLN*, *ACTA2*, and *P4HA3* (Figure 3F). *LRP1* was broadly elevated in activated fibroblast states, particularly inflammatory and profibrotic fibroblasts, but remained relatively low in homeostatic fibroblasts (Figure 3G).

### 2.4. Macrophage Subclustering Identifies Three Major Macrophage States in Keloids

**Figure 4:**
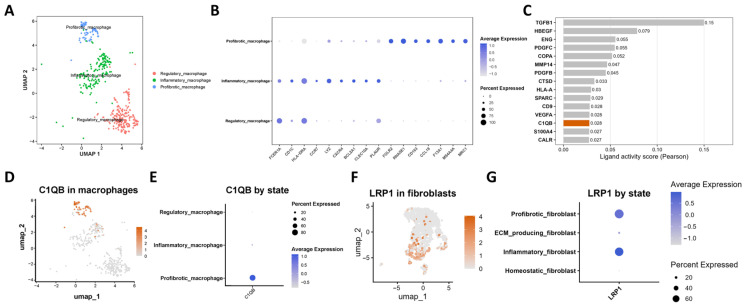
Single-cell reclustering and NicheNet analysis support a candidate C1QB^+^ profibrotic macrophage–LRP1^+^ activated fibroblast communication axis. (**A**) UMAP visualization and functional state annotation of reclustered macrophage populations; (**B**) expression patterns of representative marker genes across major macrophage functional states. Dot size indicates the proportion of positive cells, and color indicates the average expression level; (**C**) NicheNet-based ranking of candidate macrophage-derived ligands by ligand activity; (**D**) UMAP feature plot showing C1QB expression in the macrophage compartment; (**E**) comparison of C1QB expression across macrophage functional states; (**F**) UMAP feature plot showing LRP1 expression in the fibroblast compartment; (**G**) comparison of LRP1 expression across fibroblast states. Abbreviations: LRP1, low-density lipoprotein receptor-related protein 1; UMAP, uniform manifold approximation and projection.

Macrophage subclustering resolved three major transcriptional states for downstream analysis: regulatory, inflammatory, and profibrotic macrophages (Figure 4A). These results indicate that keloid-associated macrophages exhibit substantial functional heterogeneity beyond the conventional M1/M2 framework. Regulatory macrophages preferentially expressed antigen presentation-associated genes, including *FCER1A*, *CD1C*, *HLA-DRA*, and *CCR7*. Inflammatory macrophages were enriched for inflammation-associated genes, including *LYZ*, *CXCR4*, *BCL2A1*, *CLEC10A*, and *PLAUR*. Profibrotic macrophages showed high expression of *FOLR2*, *RNASE1*, *CD163*, *CCL18*, *F13A1*, *MS4A4A*, and *MRC1*, consistent with a pro-remodeling and profibrotic-supportive macrophage state with M2-like features (Figure 4B). Accordingly, profibrotic macrophages were selected as the putative sender population for subsequent macrophage–fibroblast communication analysis.

### 2.5. Integrated Single-Cell and NicheNet Analyses Support a Candidate C1QB^+^ Macrophage–LRP1^+^ Fibroblast Communication Axis

We next used NicheNet to prioritize macrophage-derived ligands predicted to regulate profibrotic fibroblast transcriptional programs. Among the ranked ligand transcripts, C1QB emerged as a biologically relevant candidate representing a C1Q-related macrophage program potentially involved in profibrotic macrophage–fibroblast communication (Figure 4C; Supplementary Table S6). Within the macrophage compartment, C1QB showed a state-enriched expression pattern, with preferential expression in profibrotic macrophages and lower expression in regulatory and inflammatory macrophages (Figure 4D,E), suggesting that profibrotic macrophages may represent a C1Q-high macrophage state and a potential source of C1Q-related paracrine signaling in keloids. On the receiver side, ligand–receptor matching nominated C1QB–LRP1 as a plausible candidate interaction at the transcriptomic-inference level. Consistent with the receptor profile defined above, the LRP1 signal was present in the profibrotic fibroblast population selected as the receiver compartment (Figure 4F,G; Supplementary Table S7). Together, single-cell state mapping, NicheNet-based ligand prioritization, and the fibrosis-associated profile of profibrotic fibroblasts support a candidate C1QB^+^ profibrotic macrophage–LRP1^+^ profibrotic fibroblast communication axis.

### 2.6. Tissue Immunofluorescence and In Vitro Phenotypic Assessment Provide Preliminary Support for a Candidate C1Q-Related Macrophage–Fibroblast Interaction in Keloids

**Figure 5:**
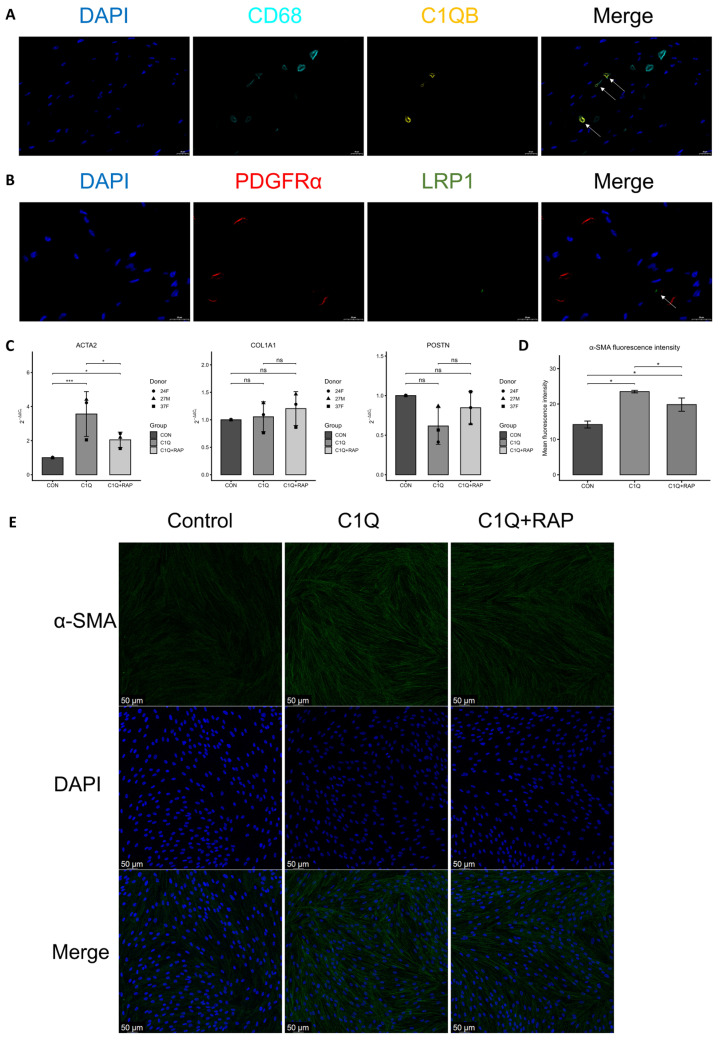
Tissue immunofluorescence and in vitro phenotypic assessment provide preliminary support for a candidate C1Q-related macrophage–fibroblast interaction involving LRP1 in keloid. (**A**) Representative immunofluorescence images showing CD68/C1QB co-staining in keloid tissue. CD68 is shown in cyan and C1QB in yellow. White arrows indicate C1QB^+^ macrophages; (**B**) representative immunofluorescence images showing PDGFRα/LRP1 co-staining in keloid tissue. PDGFRα is shown in red and LRP1 in green. White arrows indicate LRP1^+^ fibroblast-related cells; (**C**) RT-qPCR analysis of *COL1A1*, *ACTA2*, and *POSTN* expression in primary keloid fibroblasts after C1Q and C1Q + RAP treatment; (**D**) quantification of α-SMA fluorescence intensity across treatment groups; (**E**) representative immunofluorescence images of α-SMA in primary keloid fibroblasts after C1Q and C1Q + RAP treatment. Group comparisons for tissue and cellular immunofluorescence were performed using non-parametric tests; the Mann–Whitney U test was used for two-group comparisons, and the Kruskal–Wallis test was used for multi-group comparisons. For RT-qPCR analysis, group effects were evaluated using a linear mixed-effects model. Significance levels are indicated as follows: ns, not significant; * *p* < 0.05; *** *p* < 0.001. Abbreviations: LRP1, low-density lipoprotein receptor-related protein 1; PDGFRα, platelet-derived growth factor receptor alpha; RT-qPCR, reverse transcription quantitative PCR; RAP, receptor-associated protein; α-SMA, alpha-smooth muscle actin.

To obtain tissue-level and in vitro phenotypic support for this candidate interaction, we next performed experiments at the tissue and cellular levels. Tissue immunofluorescence staining showed that C1QB signals were detectable in a subset of CD68^+^ cells, and LRP1 signals were detectable in a subset of Platelet-derived growth factor receptor alpha (PDGFRα)^+^ cells in keloid (Figure 5A,B). These findings suggest CD68/C1QB and PDGFRα/LRP1 co-expression patterns consistent with the single-cell transcriptomic analysis, providing preliminary histological support for the coexistence of the predicted sender and receiver cell populations. In vitro phenotypic assessment using the intact C1Q complex showed that C1Q treatment significantly increased *ACTA2* expression in keloid fibroblasts, whereas RAP attenuated this induction. Pairwise comparisons showed higher ACTA2 expression in the C1Q group than in controls (*p* = 1.10 × 10^−4^), a residual increase in the C1Q + RAP group compared with controls (*p* = 0.033), and a significant reduction in the C1Q + RAP group compared with the C1Q group (*p* = 0.041). In contrast, *COL1A1* and *POSTN* did not show consistent statistically significant changes across groups (Figure 5C). Consistently, α-SMA immunofluorescence intensity increased after C1Q treatment and was reduced by RAP intervention. Overall differences were detected among the three groups (*p* = 0.0019), with pairwise comparisons showing increased α-SMA intensity in the C1Q group compared with controls and reduced intensity in the C1Q + RAP group compared with the C1Q group, although it remained above the control level (*p* = 0.024). Given that this immunofluorescence assay was performed using fibroblasts from a single donor, these data were interpreted as supportive phenotypic evidence (Figure 5D,E).

Together, these findings provide preliminary tissue-level and in vitro phenotypic support for a candidate C1Q-related macrophage–fibroblast interaction in keloids. Under the present experimental conditions, C1Q enhanced α-SMA-associated myofibroblast-like activation in keloid fibroblasts, and this effect was attenuated by RAP intervention. In contrast, C1Q stimulation alone did not elicit a coordinated ECM-associated transcriptional program, suggesting that C1Q-related signaling may preferentially promote an activation-prone fibroblast state rather than independently drive a fully developed matrix-producing phenotype.

## 3. Discussion

Keloids are increasingly recognized as immune–fibrotic disorders characterized by immune dysregulation and aberrant ECM remodeling. Therefore, defining how immune cells communicate with fibroblasts through specific signaling pathways is essential for understanding the mechanisms that sustain profibrotic programs in keloids [18]. Recent advances in bulk RNA-seq and single-cell RNA-seq have enabled systematic characterization of keloid-associated molecular programs at both the tissue and single-cell levels, providing new insights into the cellular heterogeneity and immune microenvironment of keloid pathology [19,20,21]. However, previous studies have mainly focused on mapping the cellular atlas of keloids, and the specific paracrine signaling axes mediating immune–stromal crosstalk, especially macrophage–fibroblast communication, remain poorly characterized. In this study, integrated transcriptomic analysis revealed that keloids exhibit not only enhanced profibrotic transcriptional programs but also sustained immune activation. Single-cell profiling further identified marked heterogeneity within both fibroblast and macrophage compartments. In particular, profibrotic fibroblast states and a C1QB-high profibrotic macrophage state emerged as candidate cellular populations that may contribute to persistent pathological remodeling. By integrating cell–cell communication inference with tissue-level observations and preliminary in vitro phenotypic evidence, we propose that C1Q-related macrophage–fibroblast communication may represent a candidate framework linking macrophage states to fibroblast activation during keloid progression.

At the bulk transcriptomic level, keloids showed enrichment of fibrosis-associated pathways together with immune-related pathways, supporting the concept that keloids are not driven solely by autonomous fibroblast dysregulation, but rather reflect aberrant tissue remodeling shaped by immune–stromal interactions [22]. The positive association between immune-related signaling and ECM/fibrosis scores further suggests that persistent keloid progression may depend, at least in part, on sustained immune-mediated modulation of fibroblast states. Because bulk RNA-seq cannot resolve the cellular origin or distribution of these programs, single-cell analysis was used to define the relevant cellular compartments at higher resolution. Consistent with previous single-cell studies showing pronounced fibroblast heterogeneity in keloids, our analysis identified multiple fibroblast states with distinct functional programs [23,24]. ECM-producing fibroblasts, marked by high expression of *COL1A1*, *COL3A1*, and *FN1*, were mainly associated with matrix synthesis and deposition, whereas profibrotic fibroblasts, characterized by *POSTN*, *ACTA2*, and related activation markers, showed stronger TGF-β response, myofibroblast activation, and profibrotic transcriptional programs. Inflammatory fibroblasts, although not the most fibrogenic population, expressed inflammatory and chemotactic mediators, suggesting that they may link immune activation with stromal remodeling. These findings indicate that maintenance of the keloid pathological phenotype is unlikely to be mediated by a single fibroblast population, but instead involves coordinated contributions from multiple functionally specialized fibroblast states. The heterogeneity of fibroblast states also implies that upstream immune regulation is likely to be state-specific. Macrophages have been closely implicated in keloid fibrosis and have been reported to exhibit M2-like features [4]. In our analysis, profibrotic macrophages expressed markers commonly associated with M2-like macrophages, including *CD163* and *MRC1*. However, fibrosis-associated macrophages are highly plastic and cannot be fully explained by the conventional M1/M2 framework [25]. Indeed, experimental keloid models suggest that distinct M2 macrophage subtypes may exert divergent effects on tissue repair, with M2a-like macrophages promoting myofibroblast differentiation and ECM remodeling, whereas M2c-like macrophages may facilitate myofibroblast dedifferentiation and resolution of repair [26]. Thus, rather than categorizing keloid-associated macrophages simply as M2-like macrophages, our findings support a more nuanced model in which a subset of macrophages acquires a pro-remodeling, profibrotic-supportive state that may sustain and amplify keloid fibrosis through paracrine signaling. This interpretation provides a rationale for investigating specific macrophage–fibroblast signaling axes.

Building on the observed macrophage heterogeneity, we focused on macrophage-derived signals that may regulate fibroblast activation. The complement system is now recognized not only as a canonical innate immune effector but also as a context-dependent regulator of tissue remodeling, repair, and fibrosis [27]. Among its components, C1Q can act beyond complement activation and participate in stromal cell regulation. In macrophages, C1QB may mark specific transcriptional states, whereas the intact C1Q complex may function as a paracrine mediator of cell–cell communication. Evidence from other fibrotic settings supports this interpretation. C1Q^+^ interstitial macrophages have been linked to the maintenance of activated fibroblasts in pulmonary fibrosis [7], and C1Q itself can activate lung fibroblasts and promote fibrotic changes [28]. Similarly, C1Q-high macrophage populations have also been described in severe alcohol-associated hepatitis and pulmonary fibrosis, where they display phagocytic, immunoregulatory, and tissue-remodeling features [8,29]. In our study, C1QB was enriched in profibrotic macrophages, and exogenous C1Q increased α-SMA expression in keloid fibroblasts, suggesting that C1QB-high macrophage states may contribute to the myofibroblast-like activation of keloid fibroblasts through C1Q-related signaling. This provides a plausible mechanism by which immune dysregulation may be translated into pathological fibroblast activation in keloids. Given the noncanonical roles of C1Q in wound healing, angiogenesis, and tissue repair, persistent C1Q-related signaling in the keloid microenvironment may represent a maladaptive extension of repair-associated signaling [30,31,32]. From a broader fibrosis perspective, this finding may indicate that macrophage-derived C1Q-related signaling occupies an upstream immune–stromal position rather than functioning as a terminal fibrogenic effector. The preferential induction of α-SMA suggests a shift toward a myofibroblast-like, contractile activation state, whereas coordinated ECM production may require additional profibrotic cues, such as mechanical tension, prolonged stimulation, or a three-dimensional matrix environment. This interpretation is consistent with the concept that macrophage-dependent stromal activation in fibrotic tissues often acts through sequential or cooperative signals rather than a single standalone pathway. It is important to distinguish C1QB as a transcriptomic marker from C1Q-related signaling as a functional concept. Our single-cell analysis identified a C1QB-high macrophage state, whereas biological effects are more likely mediated by the intact C1Q protein complex rather than the C1QB subunit alone. Therefore, we use C1QB when referring to macrophage state annotation, and C1Q-related signaling when discussing cell–cell communication, receptor interaction, and in vitro stimulation.

**Figure 6:**
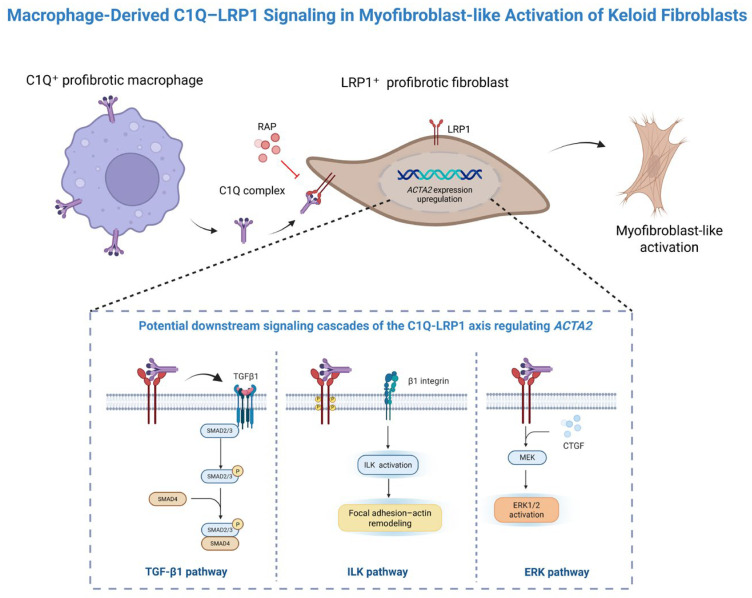
Proposed macrophage-derived C1Q–LRP1 signaling model in myofibroblast-like activation of keloid fibroblasts. C1Q^+^ profibrotic macrophages may release C1Q complexes that bind LRP1 on profibrotic keloid fibroblasts. RAP is depicted as a competitive inhibitor of LRP1-related ligand binding. C1Q–LRP1 engagement is proposed to promote *ACTA2* upregulation and α-SMA-associated fibroblast activation. The lower panel illustrates literature-supported LRP1-associated mechanisms potentially contributing to *ACTA2* upregulation, including TGF-β1 pathway, ILK pathway, and CTGF–LRP/ERK-related signaling. Dashed elements indicate putative mechanisms not directly tested in this study. Abbreviations: LRP1, low-density lipoprotein receptor-related protein 1; RAP, receptor-associated protein; ILK, integrin-linked kinase; TGF-β1, transforming growth factor beta 1; CTGF, connective tissue growth factor; ERK, extracellular signal-regulated kinase. Created in BioRender. Yuan, Y. (2026) https://BioRender.com/nx4rb1c (accessed on 20 April 2026).

In the fibroblast compartment, LRP1 expression in profibrotic fibroblasts provided a receptor-level rationale for prioritizing this population as a potential receiver of macrophage-derived C1Q-related signals. Together with NicheNet-based ligand prioritization, which identified C1QB as a candidate macrophage-derived ligand, these findings nominated the C1Q–LRP1 axis as a plausible macrophage–fibroblast communication mechanism in keloids. This prioritization is biologically supported by the known functions of LRP1 [33]. In remodeling-associated diseases, LRP1 has been implicated in fibroblast phenotypic regulation: macrophage-derived apolipoprotein E can signal through fibroblast LRP1–Extracellular signal-regulated kinase (ERK) signal to regulate matrix remodeling, whereas extracellular Heat shock protein 90 alpha (HSP90α) can act through LRP1 to promote myofibroblast persistence and expression of α-SMA and collagen 1A [11,12]. In addition, prior studies provide a mechanistic context for LRP1-associated fibroblast activation. Ligand-induced LRP1 signaling has been shown to promote LRP1 tyrosine phosphorylation, β1 integrin recruitment, and downstream integrin-linked kinase activation, thereby enhancing α-SMA and type I collagen expression in fibroblasts [34]. In parallel, connective tissue growth factor (CTGF) has been shown to engage LRP1-associated signaling, inducing LRP tyrosine phosphorylation and ERK1/2 activation, thereby potentiating TGF-β1-mediated myofibroblast activation, as reflected by enhanced α-SMA expression and fibronectin accumulation [35]. In this context, the preferential α-SMA response observed after C1Q stimulation may represent an early or amplification-related myofibroblast-like activation program, whereas a broader ECM-producing phenotype may require additional profibrotic or matrix-dependent cues. Together, these findings support the concept that LRP1-positive activated fibroblasts may act as stromal integrators of C1Q-related and other paracrine profibrotic signals. Importantly, the C1Q–LRP1 link is supported not only by computational inference but also by prior molecular evidence showing that C1Q directly binds LRP1 clusters II and IV and that this interaction can be competitively inhibited by RAP [14,15]. Consistent with this evidence, our tissue staining showed the coexistence of C1QB^+^ macrophages and LRP1^+^ fibroblasts in keloids, and in vitro assays showed that C1Q increased α-SMA expression in keloid fibroblasts, an effect attenuated by RAP. However, *COL1A1* and *POSTN* were not coordinately induced under the same conditions. Collectively, these findings support a biologically plausible model in which C1QB-high profibrotic macrophages may provide C1Q-related signals to LRP1-positive activated fibroblasts, thereby contributing to myofibroblast-like activation in keloids (Figure 6). This model integrates single-cell transcriptomic inference with tissue-level observations and preliminary in vitro phenotypic evidence, providing a focused framework for understanding macrophage–fibroblast crosstalk in keloids. Future studies integrating dedicated functional assays with three-dimensional matrix-remodeling systems may further refine this framework and define how C1Q-related macrophage–fibroblast signaling shapes fibroblast migration, contractility, and matrix-remodeling behavior.

Nevertheless, this study has several limitations. First, the number of patient-derived samples was limited, and both tissue-level validation and primary fibroblast experiments require further confirmation in larger cohorts with more independent donors. Second, the single-cell analysis identified a C1QB-high profibrotic macrophage state at the transcriptomic level, whereas the in vitro phenotypic experiments used the intact C1Q protein complex. Therefore, the current evidence supports the possible involvement of C1Q-related signaling in keloid fibroblast activation, rather than an independent functional role of the C1QB subunit alone. Third, although NicheNet inference, tissue-level coexistence of C1QB^+^ macrophages and LRP1^+^ fibroblasts, and RAP intervention collectively support the involvement of LRP1, they do not establish direct LRP1-specific dependence. Because RAP broadly inhibits ligand interactions within the LDL receptor family, contributions from other receptors or parallel signaling pathways cannot be excluded. Further in vivo and loss-of-function studies are needed to establish causality. In addition, ECM production and matrix deposition may require longer stimulation, more complex culture systems, or cooperation with other profibrotic and inflammatory cues, such as TGF-β. Thus, the C1Q–LRP1 axis is better interpreted as a potential initiating or amplifying signal for pathological fibroblast activation rather than a standalone determinant of the terminal fibrotic phenotype. Despite these limitations, this study provides a macrophage state–fibroblast receptor–pathological activation framework for understanding keloid pathogenesis. These findings refine the current understanding of the keloid immune–fibrotic microenvironment and suggest that C1Q-related macrophage–fibroblast communication may serve as a reference for future studies exploring potential intervention strategies.

## 4. Materials and Methods

### 4.1. Human Tissue Specimens and Primary Keloid Fibroblasts

This study was conducted in accordance with the Declaration of Helsinki and was approved by the institutional ethics committee of the authors’ institution (protocol code: 2026-125; date of approval: 11 May 2026). Written informed consent was obtained from all participants. For tissue immunofluorescence staining, keloid tissue was collected from an anterior chest lesion of a 27-year-old male patient. Primary keloid fibroblasts used for in vitro experiments were isolated from keloid lesions of three independent donors, including a 27-year-old male with an anterior chest keloid, a 37-year-old female with an anterior chest keloid, and a 24-year-old female with an earlobe keloid. All in vitro experiments were performed using passage 3 primary fibroblasts after isolation. Cellular immunofluorescence was performed using cells from the 27-year-old male donor, whereas reverse transcription quantitative PCR (RT-qPCR) experiments were conducted using cells from all three independent donors.

### 4.2. Bulk RNA-Seq Data Processing and Analysis

Bulk RNA-seq data were obtained from Gene Expression Omnibus (GEO) dataset GSE158395, including six normal skin samples and four keloid tissue samples. Analyses were performed using the normalized expression matrix provided by GEO in R version 4.5.2.

Principal component analysis (PCA) was performed to assess global transcriptomic variation between keloid and normal skin samples. Hierarchical clustering based on sample-to-sample Pearson correlation coefficients was used to evaluate the consistency of expression profiles across samples. Differentially expressed genes (DEGs) were identified using the limma package, with adjusted *p* < 0.05 and |log_2_ fold change| > 1 as thresholds. DEGs were visualized using volcano plots, and the top 50 DEGs ranked by adjusted *p* value were displayed in a heatmap with row-scaled expression values. Functional enrichment analysis of DEGs was performed for Gene Ontology (GO) terms and KEGG pathways using clusterProfiler. For KEGG analysis, gene symbols were converted to Entrez IDs using org.Hs.eg.db, and pathway annotation was performed using the human KEGG organism code “hsa”. Genes were ranked by the differential expression statistic, and GSEA was performed to evaluate pathway-level alterations between keloid and normal skin samples. Sample-level ECM/fibrosis and immune-related signaling scores were calculated as the mean expression of predefined ECM/fibrosis and immune-related gene signatures, respectively. Between-group comparisons were performed using the Wilcoxon rank-sum test, and correlations between signature scores were assessed using Spearman correlation analysis.

### 4.3. Single-Cell RNA-Seq Processing and Cell-Type Annotation

Single-cell RNA-seq data were obtained from GEO dataset GSE282885, including three keloid and three normal skin samples. All analyses were performed in R version 4.5.2 using Seurat. Raw count matrices were imported into R, and cells were retained using the following quality-control criteria: 300 < nFeature_RNA < 6000 and percent.mt < 20%. A Seurat object was generated for each sample, and all objects were merged for downstream analysis.

The merged object was normalized using LogNormalize with a scale factor of 10,000. The top 2000 highly variable features were identified, followed by data scaling and PCA. Based on the ElbowPlot, the first 20 principal components were used for nearest-neighbor graph construction, unsupervised clustering, and UMAP visualization. The clustering resolution for the global analysis was set to 0.5. Cluster-enriched marker genes were identified using FindAllMarkers with min.pct = 0.1, logfc.threshold = 0.25, and only.pos = TRUE. Major cell types were annotated according to cluster-enriched markers and canonical lineage markers.

Fibroblasts were then subsetted from the integrated object for focused reanalysis. After removal of clusters with clear non-fibroblast marker expression, fibroblasts were subclustered and annotated into distinct functional states. To characterize state-specific functional programs, module scores were calculated using AddModuleScore in Seurat. Gene Ontology biological process enrichment and Hallmark gene set analyses were further performed to define functional features of fibroblast states. Expression patterns of representative genes and candidate receptors were visualized using DotPlot and FeaturePlot.

### 4.4. Macrophage Subclustering and State Annotation

Macrophages were subsetted from the integrated single-cell object for dedicated subcluster analysis. After removal of clusters showing clear non-macrophage signatures, the macrophage subset was reprocessed, including normalization, dimensionality reduction, clustering, and state annotation. For macrophage subclustering, the first 15 principal components were used for nearest-neighbor graph construction and UMAP visualization, with the clustering resolution set to 0.4. State-enriched marker genes were identified by differential expression analysis and interpreted together with canonical macrophage markers reported in the literature. Representative marker expression across macrophage states was visualized using DotPlot.

### 4.5. Inference of Macrophage–Fibroblast Communication

To identify signaling programs potentially involved in immune–fibrotic crosstalk in keloids, ligand–receptor–target relationships between macrophages and fibroblasts were inferred using the NicheNet framework. Profibrotic macrophages were defined as the sender population, and profibrotic fibroblasts were defined as the receiver population. Candidate macrophage-derived ligands were prioritized according to ligand activity, which estimates the ability of each ligand to predict fibrosis-associated target gene programs in the receiver population. Ligands were ranked by Pearson correlation coefficients derived from NicheNet ligand activity prediction.

Candidate ligands with high biological interpretability and limited prior characterization in keloids were selected for downstream analysis. To further evaluate candidate communication axes, ligand expression across macrophage states and corresponding receptor expression across fibroblast states were jointly assessed and visualized. In DotPlot visualizations, dot size represents the proportion of cells expressing the indicated gene, whereas color intensity represents the average normalized expression level.

### 4.6. Tissue Immunofluorescence Staining

Tissue immunofluorescence staining was performed to validate the presence of C1QB^+^ macrophages and LRP1^+^ fibroblasts in keloid tissue. Keloid samples were fixed in 4% paraformaldehyde, paraffin-embedded, and sectioned at 4 μm. After deparaffinization, rehydration, and heat-induced antigen retrieval in Tris–EDTA buffer (pH 8.0), sections were blocked with 5% bovine serum albumin (BSA). Sections were incubated with primary antibodies overnight at 4 °C, followed by species-matched fluorophore-conjugated secondary antibodies for 1 h at room temperature in the dark. Nuclei were counterstained with DAPI, and representative images were acquired using a Leica fluorescence microscope (Leica Microsystems GmbH, Wetzlar, Germany) under identical acquisition settings. Co-expression was qualitatively assessed based on overlapping fluorescence signals in representative fields. Antibody information, fluorophores, imaging channels, and dilution ratios are listed in Supplementary Table S1.

### 4.7. Primary Keloid Fibroblast Isolation, Culture, and Treatment

Primary keloid fibroblasts were isolated using the tissue explant method, which is commonly used for primary keloid fibroblast culture. Fresh keloid specimens were processed under sterile conditions, rinsed repeatedly with prechilled PBS to remove residual blood, and dissected to remove the epidermis and subcutaneous adipose tissue. The remaining dermal/keloid tissue was minced into approximately 1-mm^3^ fragments and placed onto culture dishes to allow tissue adherence. After tissue attachment, Dulbecco’s modified Eagle medium (DMEM) supplemented with 10% fetal bovine serum and 1% penicillin–streptomycin was added carefully. Explants were maintained at 37 °C in a humidified incubator with 5% CO_2_, and the medium was replaced every 2–3 days. Cells were passaged at 80–90% confluence, and passage 3 primary keloid fibroblasts were used for subsequent experiments.

Based on previously reported effective concentration ranges, cells were assigned to three groups: control, C1Q, and C1Q + RAP. C1Q (A099; Complement Technology, Inc., Tyler, TX, USA) was used at a final concentration of 10 μg/mL, and RAP (HY-P76478; MedChemExpress LLC, Monmouth Junction, NJ, USA) was used at 20 μg/mL. These concentrations were selected based on previous in vitro studies showing that C1Q at 10 μg/mL can elicit C1Q-related signaling responses, while RAP at 20 μg/mL with short-term pretreatment has been used as an LDL receptor family antagonist to interfere with LRP1-related ligand binding [28,34]. In the C1Q + RAP group, cells were pretreated with RAP for 1 h before C1Q stimulation and then cultured for an additional 48 h. Control cells received an equal volume of culture medium. After treatment, cells were collected for immunofluorescence staining and RT-qPCR analysis.

### 4.8. RNA Extraction, Reverse Transcription, and RT-qPCR

Total RNA was extracted from treated cells using TRIzol reagent, and RNA concentration and purity were assessed before reverse transcription. cDNA was synthesized using HiScript IV All-in-One Ultra RT SuperMix for qPCR (R433-01; Vazyme Biotech Co., Ltd., Nanjing, China) according to the manufacturer’s instructions. Reverse transcription quantitative PCR (RT-qPCR) was performed on a LightCycler 480 System (Roche Diagnostics GmbH, Mannheim, Germany) using LightCycler 480 SYBR Green I Master (Roche Diagnostics GmbH, Mannheim, Germany). The cycling conditions were as follows: 95 °C for 5 min, followed by 45 cycles of 95 °C for 10 s, 60 °C for 30 s, and 72 °C for 20 s. Melting curve analysis was performed to verify amplification specificity. The expression levels of *COL1A1*, *ACTA2*, and *POSTN* were measured, with β-actin used as the reference gene. RT-qPCR was performed using primary fibroblasts from three independent donors. For each donor, three independent experiments were conducted, with three technical replicates for each treatment condition in each experiment. Primer sequences are listed in Supplementary Table S1.

### 4.9. Immunofluorescence Staining of Cultured Cells

Cellular immunofluorescence staining was performed to assess treatment-induced phenotypic changes in primary keloid fibroblasts. Cells were seeded on sterile coverslips in 6-well plates. After treatment, cells were fixed with 4% paraformaldehyde for 15 min at room temperature, permeabilized with 0.1% Triton X-100 for 15 min, and blocked with 5% BSA for 1 h. Cells were incubated with an anti-alpha-smooth muscle actin (α-SMA) primary antibody overnight at 4 °C, followed by incubation with an Alexa Fluor 488-conjugated goat anti-rabbit secondary antibody for 1 h at room temperature in the dark. Nuclei were counterstained with DAPI, and coverslips were mounted for fluorescence imaging. Images were acquired under identical exposure settings across groups, and α-SMA fluorescence intensity was quantified using ImageJ (version 1.52a). Antibody sources, catalog numbers, and dilution ratios are listed in Supplementary Table S1.

### 4.10. Image Quantification and Statistical Analysis

For cellular immunofluorescence, mean fluorescence intensity was quantified across multiple representative fields per condition using primary keloid fibroblasts from a single donor; therefore, these data were interpreted as supportive phenotypic evidence. For immunofluorescence quantification, the Mann–Whitney U test was used for two-group comparisons, and the Kruskal–Wallis test was used for comparisons among three or more groups. For RT-qPCR analysis, Ct values from technical triplicates within each independent experiment were averaged, and ΔCt values were calculated using β-actin as the reference gene. Treatment effects were assessed on ΔCt values using a linear mixed-effects model, with treatment group as a fixed effect and donor and independent experiment nested within donor as random effects. For visualization, ΔΔCt values were calculated using the corresponding control condition from the same donor and independent experiment as the calibrator, and relative expression was presented as 2^−ΔΔCt. Statistical analyses were performed using R version 4.5.2. All tests were two-sided, and *p* < 0.05 was considered statistically significant.

## 5. Conclusions

This study integrates bulk transcriptomic profiling, single-cell analysis, and preliminary in vitro assays to characterize the immune–fibrotic microenvironment of keloids. Our findings revealed coordinated immune activation and fibrotic remodeling at the tissue level, together with marked functional heterogeneity of fibroblasts and macrophages at single-cell resolution. In particular, a C1QB-high profibrotic macrophage state and LRP1-positive activated fibroblast states were identified as candidate cellular components of macrophage–fibroblast crosstalk in keloids. Tissue-level observations and in vitro phenotypic assessment further provided preliminary support for a candidate C1Q-related macrophage–fibroblast interaction, which may contribute to α-SMA-associated myofibroblast-like activation of keloid fibroblasts. Overall, these findings provide a preliminary framework for understanding immune-driven fibroblast activation during keloid progression and may serve as a reference for future studies exploring potential intervention strategies.

## Data Availability

The publicly available datasets analyzed in this study are available from the Gene Expression Omnibus database under accession numbers GSE158395 and GSE282885. The experimental data generated during the present study, including RT-qPCR and immunofluorescence data, are available from the corresponding author upon reasonable request.

## Supplementary Materials

The following supporting information can be downloaded at: https://www.mdpi.com/article/10.3390/ijms27115140/s1.

## Abbreviations

The following abbreviations are used in this manuscript:

α-SMA	Alpha-smooth muscle actin
BSA	Bovine serum albumin
CTGF	Connective tissue growth factor
DEG	Differentially expressed gene
DMEM	Dulbecco’s modified Eagle medium
ECM	Extracellular matrix
ERK	Extracellular signal-regulated kinase
GEO	Gene Expression Omnibus
GO	Gene Ontology
GSEA	Gene set enrichment analysis
HSP90α	Heat shock protein 90 alpha
KEGG	Kyoto Encyclopedia of Genes and Genomes
LDL	Low-density lipoprotein
LRP1	Low-density lipoprotein receptor-related protein 1
NES	Normalized enrichment score
PBS	Phosphate-buffered saline
PCA	Principal component analysis
PDGFRα	Platelet-derived growth factor receptor alpha
RAP	Receptor-associated protein
RT-qPCR	Reverse transcription quantitative polymerase chain reaction
TGF-β	Transforming growth factor beta
UMAP	Uniform manifold approximation and projection
