# Supplementary material for: Integrated Transcriptomic Analysis Suggests a C1Q-Related Macrophage–Fibroblast Signaling Axis in Keloids
Source: Int J Mol Sci. 2026 Jun 5;27(11):5140. doi: 10.3390/ijms27115140 (PMC13257865; doi:10.3390/ijms27115140)
Supplement: Supplementary file 1 [file ijms-27-05140-s001.zip › Supplementary Figure S1.pdf]

Figure S1. Supplementary bulk RNA-seq functional enrichment analysis, single-cell RNA-seq quality control, and functional enrichment of fibroblast states.

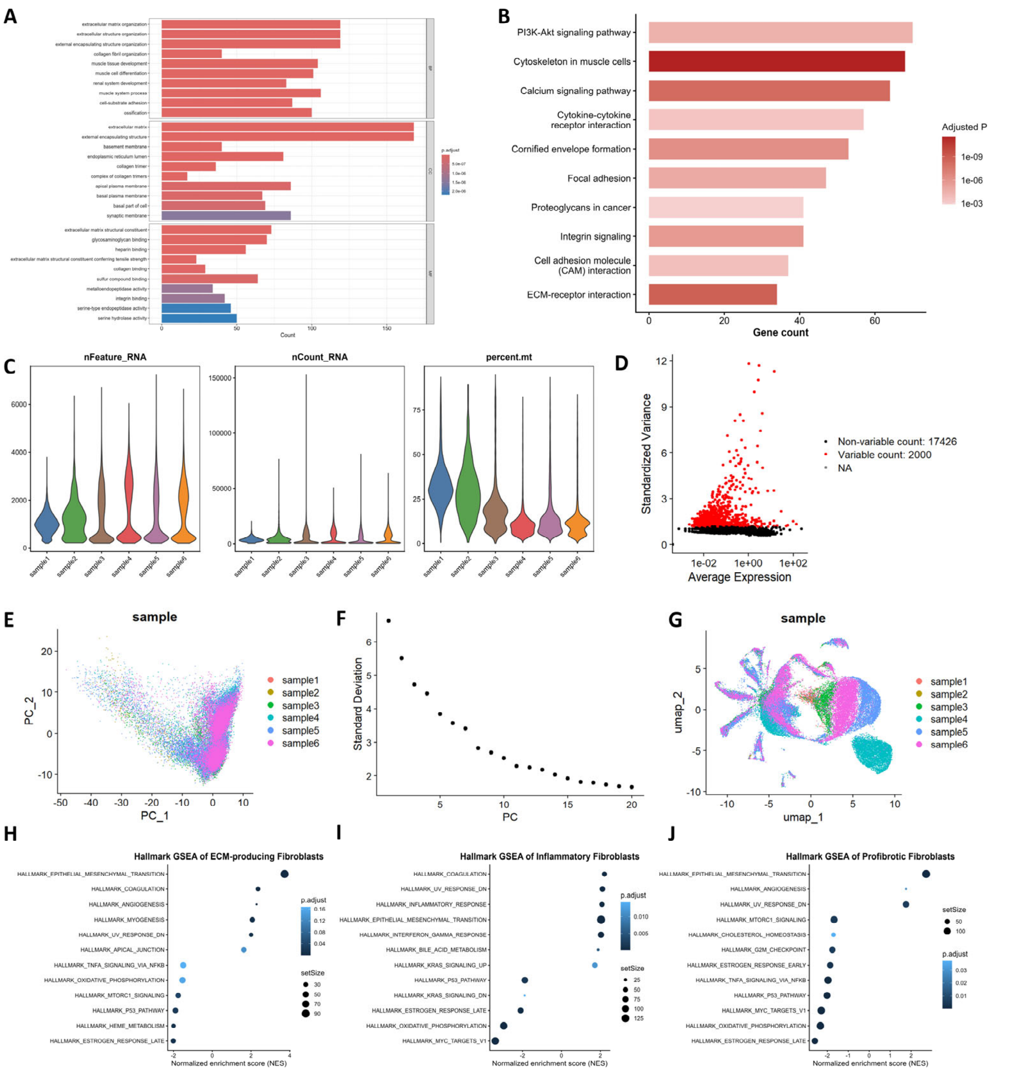

Figure legend: (a) GO enrichment analysis of differentially expressed genes between keloid tissue and normal skin. Enriched terms are grouped into BP, CC, and MF categories. The x-axis indicates the number of differentially expressed genes included in each GO term, and the color scale indicates the adjusted P value; (b) KEGG pathway enrichment analysis of differentially expressed genes. The x-axis indicates the number of differentially expressed genes included in each pathway, and the color scale indicates the Benjamini–Hochberg-adjusted P value; (c) Quality control of scRNA-seq data. Violin plots show the distributions of nFeature_RNA, nCount_RNA, and percent.mt across six samples. Cells with 300 < nFeature_RNA < 6000 and percent.mt < 20% were retained for downstream analyses; (d) Identification of HVGs. Red dots indicate the 2,000 selected

HVGs, whereas black dots indicate non-variable genes. The selected HVGs were used for subsequent PCA, cell clustering, and dimensionality reduction; (e) PCA based on HVGs. Cells are colored by sample origin to evaluate the overall distribution of individual samples in low-dimensional transcriptomic space; (f) ElbowPlot for PC selection. Based on the distribution of standard deviations across PCs, the first 20 PCs were selected for nearest-neighbor graph construction, cell clustering, and UMAP visualization; (g) Integrated UMAP visualization of the single-cell atlas colored by sample origin. Cells from different samples were well mixed after integration, indicating no obvious batch effect; (h) Hallmark gene set enrichment analysis of differentially expressed genes associated with Inflammatory_fibroblast. This fibroblast state was mainly enriched for inflammatory response, stress-response, and partial stromal activation programs; (i) Hallmark gene set enrichment analysis of differentially expressed genes associated with ECM_producing_fibroblast. This fibroblast state was primarily enriched for ECM remodeling, stromal activation, and tissue remodeling programs; (j) Hallmark gene set enrichment analysis of differentially expressed genes associated with Profibrotic_fibroblast. This fibroblast state exhibited prominent profibrotic activation, stress-response, and metabolic reprogramming features. In Hallmark enrichment plots, the x-axis represents NES, dot size indicates gene set size, and color indicates the adjusted P value. Abbreviations: BP, biological process; CC, cellular component; ECM, extracellular matrix; GO, Gene Ontology; HVGs, highly variable genes; KEGG, Kyoto Encyclopedia of Genes and Genomes; MF, molecular function; NES, normalized enrichment score; PC, principal component; PCA, principal component analysis; scRNA-seq, single-cell RNA sequencing; UMAP, uniform manifold approximation and projection; UMI, unique molecular identifier.
